# Uncertainties of potentials and recent changes in global yields of major crops resulting from census- and satellite-based yield datasets at multiple resolutions

**DOI:** 10.1371/journal.pone.0203809

**Published:** 2018-09-20

**Authors:** Toshichika Iizumi, Mizuki Kotoku, Wonsik Kim, Paul C. West, James S. Gerber, Molly E. Brown

**Affiliations:** 1 Institute for Agro-Environmental Sciences, National Agriculture and Food Research Organization (NARO), Tsukuba, Ibaraki, Japan; 2 Graduate School of Life and Environmental Sciences, University of Tsukuba, Tsukuba, Ibaraki, Japan; 3 Institute on the Environment (IonE), University of Minnesota, St Paul, Minnesota, United States of America; 4 Department of Geographical Sciences, University of Maryland, College Park, Maryland, United States of America; Institute of Genetics and Developmental Biology Chinese Academy of Sciences, CHINA

## Abstract

Global agriculture is under pressure to meet increasing demand for food and agricultural products. There are several global assessments of crop yields, but we know little about the uncertainties of their key findings, as the assessments are driven by the single best yield dataset available when each assessment was conducted. Recently, two different spatially explicit, global, historical yield datasets, one based on agricultural census and the other largely based on satellite remote sensing, became available. Using these datasets, we compare the similarities and differences in global yield gaps, trend patterns, growth rates and changes in year-to-year variability. We analyzed maize, rice, wheat and soybean for the period of 1981 to 2008 at four resolutions (0.083°, 0.5°, 1.0° and 2.0°). Although estimates varied by dataset and resolution, the global mean annual growth rates of 1.7–1.8%, 1.5–1.7%, 1.1–1.3% and 1.4–1.6% for maize, rice, wheat and soybean, respectively, are not on track to double crop production by 2050. Potential production increases that can be attributed to closing yield gaps estimated from the satellite-based dataset are almost twice those estimated from the census-based dataset. Detected yield variability changes in rice and wheat are sensitive to the choice of dataset and resolution, but they are relatively robust for maize and soybean. Estimates of yield gaps and variability changes are more uncertain than those of yield trend patterns and growth rates. These tendencies are consistent across crops. Efforts to reduce uncertainties are required to gain a better understanding of historical change and crop production potential to better inform agricultural policies and investments.

## Introduction

The demand for crops is anticipated to double by 2050, putting more pressure on global agriculture to achieve the production goal [1]. Climate change has already added burdens for crop production [2]. To address these challenges, several studies have presented global assessments of the yield gaps [3], yield trend patterns [4], yield growth rates [5] and changes in year-to-year yield variability [6]. However, we know little about the uncertainties in the estimated potentials and changes in global yields because they were driven by the single best available yield dataset when each study was conducted. An uncertainty analysis comparing different datasets is available for yield gaps [7] and other variables [8]. Understanding which countries have production potentials and/or difficulties in recent yield growth and stabilization, with uncertainty due to multiple datasets, will help improve government agricultural policies as well as investment strategies.

For earlier assessments, either of two different global, historical yield datasets is utilized. One is compiled by Ray et al. [4] (referred to as the R12 dataset), which is a crop yield and area harvested database covering ~2.5 million statistics in ~13,500 political units globally from 1961 to 2008. The other is described by Iizumi et al. [9] (referred to as the I14 dataset) and is a hybrid of national yield statistics reported by the Food and Agriculture Organization of the United Nations (FAO) and a satellite-derived crop-specific vegetation index. The I14 dataset initially covered the period 1982 to 2006 with the grid size of 1.125° and extended to cover the period 1981 to 2011 [6]. Both datasets cover four major crops–maize, rice, wheat and soybean–which together account for two-thirds of the world’s food calories.

The datasets have their respective advantages and limitations. For instance, although grid-cell yield data are available in the R12 dataset, their spatial representativeness is not uniform across grid cells because data in some regions are based on national statistics, whereas those in other regions are based on subnational statistics. The extent of area is largely different from one political unit (or census unit) to another. In contrast, the I14 dataset is thought to have more “consistent” spatial representativeness of yields across grid cells owing to the use of satellite data. However, grid-cell yields in the I14 dataset are modeled data and those in minor crop-producing regions could be less reliable than in major crop-producing regions due to the inherent limitations of satellite remote sensing in capturing crop status in areas where crop is sparsely grown. The resolution of the I14 dataset is higher than that of the R12 dataset when the R12 dataset uses national census data, although the quality of grid-cell yield data of the I14 dataset varies by the extent of cropland within a grid cell. Therefore, a consistent result across the two datasets improves the reliability of assessment results.

It is therefore useful to address whether findings of earlier studies derived from one single dataset are robust when another dataset is analyzed. Such a practice improves our understanding of uncertainties in estimated potentials and changes in global yields. Furthermore, the difference in spatial resolution is an important aspect that differentiates the datasets. It is well documented that use of yield datasets with different resolutions often leads to different conclusions [10, 11]. We therefore compared the datasets at four different resolutions (0.083°, 0.5°, 1.0° and 2.0°). A simple comparison of the datasets is in part presented in Elliott et al. [12], but no in-depth comparison has yet conducted. We present the first assessment using the two datasets at several resolutions to examine whether the findings of earlier studies on yield gaps [3], trend patterns [4], growth rates [5] and variability changes [6] are robust when using different datasets and resolutions.

## Materials and methods

### Yield datasets

Two yield datasets–the R12 dataset [4] and an updated version of the I14 dataset [6, 9]–were used. The I14 dataset covers from 1981 to 2011, whereas the R12 dataset covers from 1961 to 2008. Our intercomparison thus uses the data for the common 28-yr period (1981 to 2008). The spatial aggregation was conducted for each dataset to provide coarser-resolution datasets at the 0.5°, 1° and 2° resolutions from the 0.083°-resolution datasets. See text S1 for details of the 0.083°-resolution I14 dataset. Historical harvested area was considered as the weight for the R12 dataset when conducting the spatial aggregation, whereas the harvested area in 2000 [13] was used for the I14 dataset throughout the studied period.

### Methods

Yield gaps were estimated according to the method of Mueller et al. [3]. Yield trend patterns and growth rates were categorized according to the method of Ray et al. [4] and Ray et al. [5], respectively. Changes in yield variability were classified as by Iizumi and Ramankutty [6]. Details are given below.

#### Yield gaps

Yield gaps are estimated as a percentage of attainable yield achieved circa the year 2000 (1997–2003). Attainable yields are assumed as the upper limit of yields likely achievable using current agronomic technology and management. They are more conservative than biophysical limits of yields and are determined by identifying high-yielding areas within bins of similar climate [3].

We recalculated the attainable yields for the R12 and I14 datasets because they seemed to be sensitive to the yield data used for the calculation, and the attainable yields used in Mueller et al. [3] were calculated based on M3-Crops data [13]. We first calculated the average yields in 1997–2003 to represent actual yields circa the year 2000. Then, grid-cell yields across the world located within the same climate bin were collected, and the value of the 95^th^ percentile of the data was used as the attainable yield for a given climate bin. This calculation was repeated from one climate bin to another to complete all climate bins in which a crop of interest is harvested. The calculated attainable yields at a resolution of 0.083° were aggregated to coarser resolutions using the harvested areas in 2000 [13] as the weights to estimate yield gaps at coarser resolutions. The climate bin definitions used here are identical to those of Mueller et al. [3]. The yield gaps presented in Mueller et al. [3] are shown as a reference in our intercomparison. Those calculated using the spatial production allocation model (SPAM) yield data [14] are also used as a reference.

#### Yield trend patterns

Ray et al. [4] categorizes global yield trends into four patterns: (1) yield never improved–areas that have witnessed no significant yield increases to date; (2) yield stagnated–areas where yields previously improved but are stagnating or declining over recent years; (3) yields collapsed–areas where yields decreased since the beginning of a studied period or initially increased and then decreased to the starting level in that period; and (4) yields still increasing–areas where yields increased at various rates throughout the time period. The last category was further divided into three subcategories, yields increasing rapidly, yields increasing moderately and yields increasing slowly, based on average yield growth rates in 1981–2008.

The distinction between these yield trend patterns is guided by the type of regression model selected. Although we followed the method described by Ray et al. [4], our analysis used a shorter yield time series than Ray et al. [4]. We fitted an intercept-only model, linear model, quadratic model and cubic model to the yield time series at each grid cell and selected a single model that fit the best to the data in terms of the Akaike Information Criterion. If an intercept-only model fit best, it indicates that yields never improved. A linear model with positive slope indicates that yields are still increasing, whereas a linear model with negative slope shows that yields collapsed. If the selected model was quadratic, a positive quadratic term indicates that yields are increasing. In contrast, a negative quadratic term indicates that the yield trend has a year at which the yield peaked. When the year at which the yield peaked was beyond the year 2010, it indicates yields are still increasing. However, the model indicates that yields are stagnating when the year at which the yield peaked was 2010 or before. Furthermore, the pattern could be recategorized to be yields collapsed if the mean yield in 2001–2008 was lower than that in 1981–1990. Although Ray et al. [4] compared yields in the 1960s and 2000s, we used yields in the 1980s in this study instead of those in the 1960s. If the selected model was a cubic model, we calculated the year when yield improvement started (i.e., the inflection point) as well as the year of yield maximum using yields for 1981–2012 estimated using the regression curve. When the year at which the yield peaked was 2010 or before, it indicates that yields are stagnating. When the yield peaked after 2010, we classified those areas as yields are increasing. As in the quadratic case, when the average yield in the 2000s was lower than that in the 1980s, we classified those areas as yields collapsed. Although different categories of yield trend patterns were proposed by Grassini et al. [15], the purpose of this study is to repeat the analysis of earlier studies using different datasets and resolutions; thus, we followed the categories used by Ray et al. [4] as closely as possible.

#### Yield growth rates

The percentage rates of average yield growth for the past 20 years (1989–2008) were calculated by fitting a linear regression curve to yield a time series over that period, as was done by Ray et al. [5], for each dataset and resolution. Then, we calculated possible production increases by 2050, relative to 2008, from the recent growth rates. We used the growth rates presented by Ray et al. [5] as a reference.

#### Yield variability changes

Although an earlier study [6] used combinations of two different yield detrending methods and two different measures of yield variability change to take methodological uncertainty into account, we selected a single combination for this study. This is because the methodological uncertainty is rather small in most cases [6], and our focus here is to reveal the similarities and differences between different datasets and resolutions. The detrending method used here is a 5-yr running mean, and the measure used is the slope of a linear regression curve fitted to a 9-yr moving window standard deviation (SD) time series of percentage yield anomalies. Changes in the yield variability were categorized into four patterns: (1) yield variability significantly increased, (2) yield variability insignificantly increased, (3) yield variability significantly decreased and (4) yield variability insignificantly decreased. The significance was estimated using bootstrap resampling over all samples with a 5% confidence level, as was done by Iizumi and Ramankutty [6]. The yield variability changes presented in an earlier study [6] are used as a reference.

## Results

### Yield gaps

**Fig 1:**
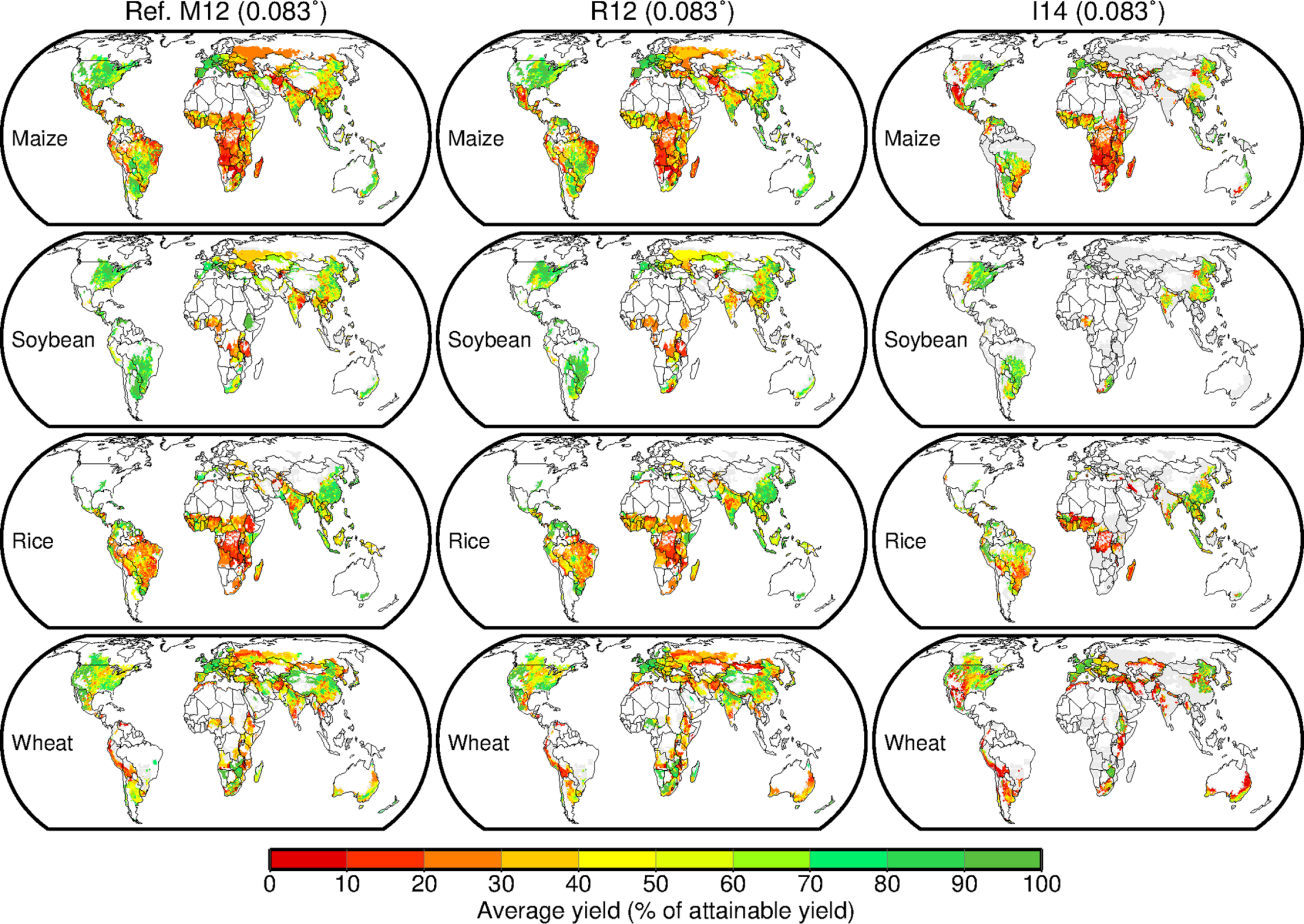
Average yields circa the year 2000 for four crops (indicated as a percentage of the attainable yield) calculated using the two yield datasets at 0.083° resolution. Data presented by Mueller et al. [3] are shown as a reference (Ref. M12). Another reference calculated based on SPAM data (Ref. SPAM) is available in Figure A in S1 File. The gray shaded area indicates that a crop of interest is harvested, but attainable yield data are not defined according to the methods of Mueller et al. [3].

For the four crops, the major geographical patterns of average yields (represented by a percentage of attainable yield) calculated using the R12 and I14 datasets showed a close match with the references [3, 14] and each other (Fig 1 and Figure A in S1 File). However, differences between the datasets were found for some regions. For maize, such regions included the western part of the United States (US) and the northern part of China. The average yields in the US were 87% and 63% of the attainable yields in the R12 and I14 dataset, respectively (Figure B in S1 File). The corresponding values were 54% and 49% in China. For soybean, rice and wheat, a similar level of correspondence with maize was found, although contrasting results between the datasets appeared for rice in Japan (the country average yield was 91% and 32% of the attainable yields in the R12 and I14 dataset, respectively) and the central part of China (84% and 56%) and for wheat in the western part of the US (56% and 48%), the northwestern part of Mexico (81% and 44%) and the northeastern part of China (71% and 53%). These contrasts were persistent across the resolutions but diminished to some degree when the spatial aggregation was conducted (Figure C in S1 File).

Differences in global production increases from closing yield gaps to 100% of attainable yields between the two datasets were surprisingly large. The production increases calculated using the I14 dataset were almost double of those derived from the R12 dataset (Table 1). We found that the empirical cumulative distributions of average yields calculated using the I14 dataset had longer tails than those computed from the R12 dataset and references (Figure D in S1 File). Attainable yields (represented by the value of the 95^th^ percentile of the grid-cell yields per climate bin) calculated using the I14 dataset were thus far larger than those calculated using the R12 dataset. As a result, the percentages of average yields for the globes and major producers, relative to attainable yields, estimated using the I14 dataset were almost always smaller than those estimated using the R12 dataset (Figure B in S1 File). The differences in resolution played a minor role in the uncertainty of the yield gaps.

**Table 1:** Production increases from closing yield gaps.

Crop	% of production in 2000			
	References		R12	I14
	M12 [3]	SPAM		
Maize	64	77	61–65	111–114
Soybean	25	44	32–33	68–70
Rice	47	77	44–47	113–124
Wheat	71	92	89–93	142–149

### Yield trend patterns

**Fig 2:**
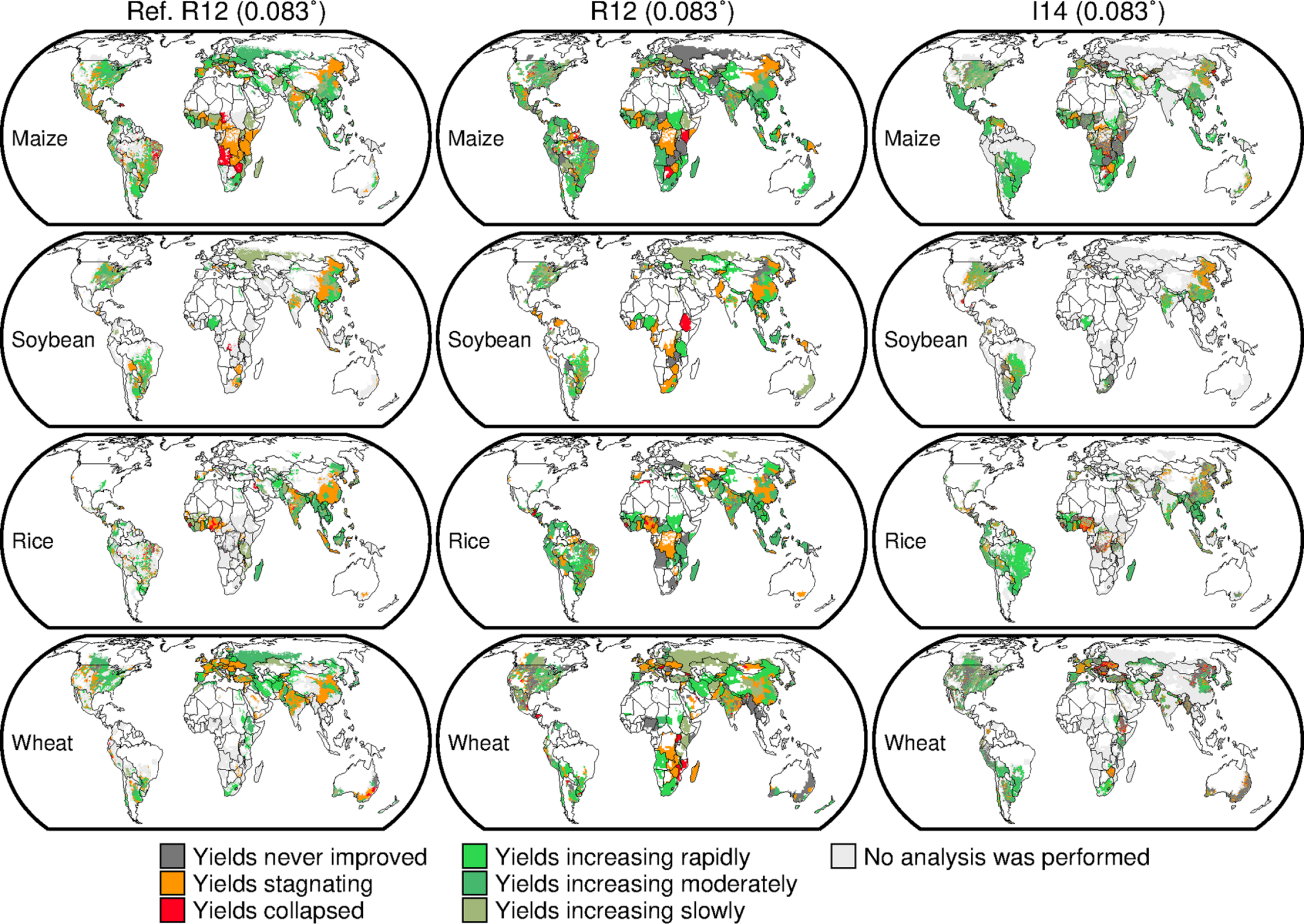
Yield trend patterns of four crops in 1981–2008 calculated using the two datasets at 0.083° resolution. Those presented by Ray et al. [4] are for the period 1961–2008 and are used as a reference (Ref. R12). The data were divided into the six yield trend patterns and color coded. The gray shaded area indicates that a crop of interest was harvested but yield data are lacking.

The major geographical characteristics of yield trend patterns for the four crops calculated using the I14 dataset are similar (Fig 2). This tendency was consistent across the different resolutions (Figure E in S1 File). Good agreement between the datasets was found for major producers in most cases, including maize and soybean in the US, Brazil and Argentina; rice in India; and wheat in the US, France and Australia. However, differences between the datasets emerged for some major producers, such as China. In the 0.083°-resolution datasets, wheat yields over the northeastern part of China increased rapidly in the R12 dataset, but the I14 dataset showed that yields in that region stagnated, never improved or even collapsed (Fig 2). In addition, the R12 dataset showed that wheat yields stagnated over Ukraine, whereas the I14 dataset depicted contrasting yield trend patterns between the western part (yields collapsed) and eastern part (yields stagnated) of that country. For soybean, the R12 dataset showed that yields in South Africa stagnated or increased rapidly, whereas the I14 dataset depicted that yields in most parts of that country never improved. Yield trend patterns derived from the R12 dataset clearly varied from one political unit to another (that is, country in this case), as seen for maize in Africa, whereas the I14 dataset showed more detailed geographic pattern within countries (Fig 2).

Ray et al. [4] conclude that, although yields of the four crops continue to increase in many areas, yields across 24–39% of maize-, soybean-, rice- and wheat-growing areas are not improving, stagnated or have collapsed. Our analysis corroborated that yields are increasing in many more areas than where they are not increasing, although the extent of areas varied by dataset and resolution in absolute terms (Table 2). For instance, the wheat-growing areas where yields are not increasing (33–36%) were comparable to those where yields are increasing (35–54%) in the I14 dataset at finer resolutions. However, in the R12 dataset, the former areas (60–61%) were always larger than the latter areas (39–40%) regardless of the resolution.

**Table 2:** Areas where yields are increasing and not increasing estimated using two different yield datasets.

Crop	% of the harvested area in 2000					
	Reference [4]		R12		I14	
	NI^a^	IM^b^	NI	IM	NI	IM
Maize	30	70	25–29	68–75	21–23	64–70
Soybean	24	76	17–21	71–83	21–24	68–74
Rice	38	63	32–33	66–68	22–28	53–67
Wheat	39	61	39–40	60–61	33–36	35–54

^a^ Yields not improving (= yields never improved + yields stagnating + yields collapsed).

^b^ yields improving (= yields increasing rapidly + yields increasing moderately + yields increasing slowly).

### Yield growth rates

**Fig 3:**
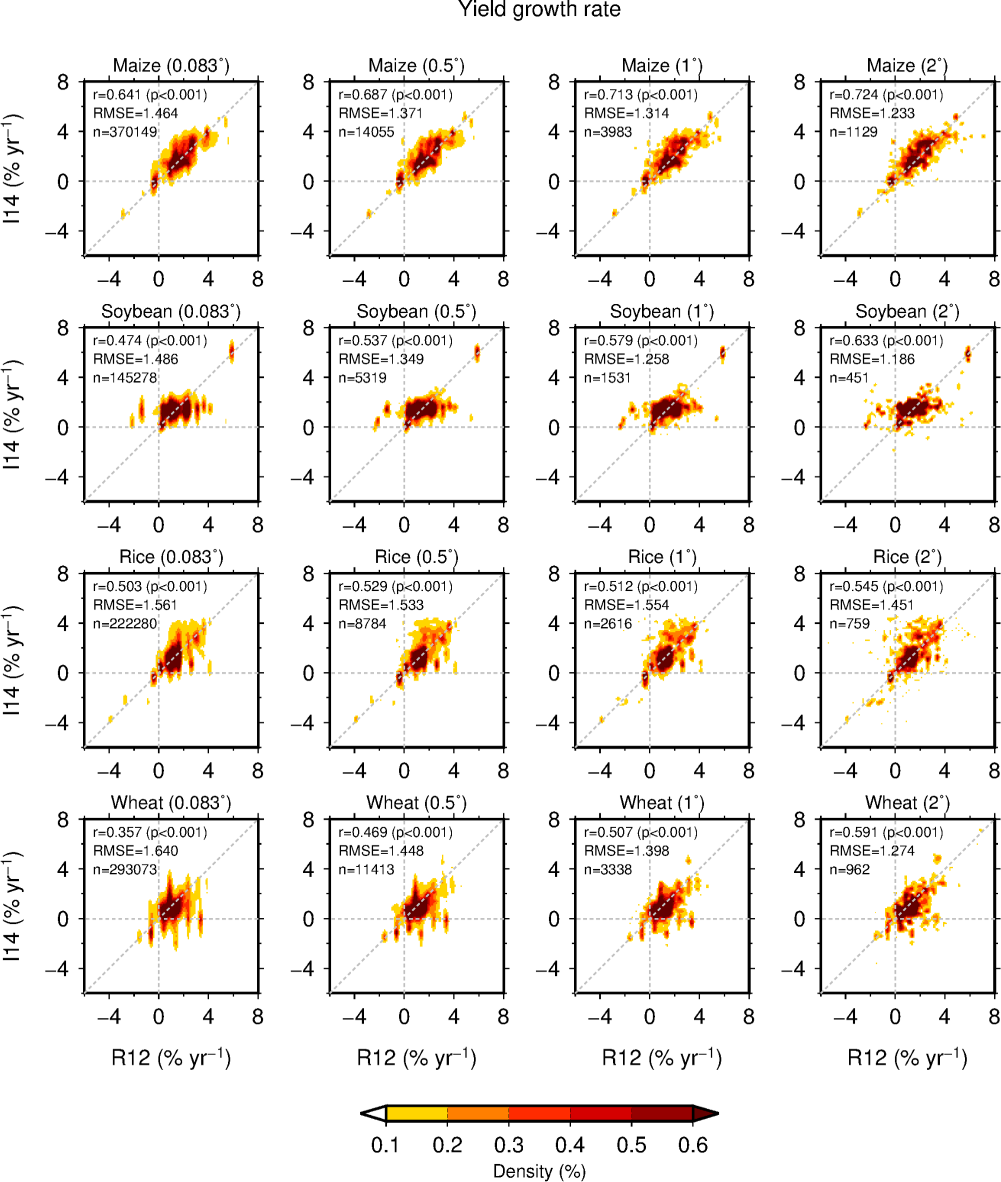
The correspondence in grid-cell average annual rates of yield growth in 1989–2008 for four crops calculated using the two datasets at four resolutions. The colored shaded area shows the smoothed density of the grid-cell data. The correlation coefficient (r), p-value (p), root-mean-squared error (RMSE) and sample size (n) are presented.

The uncertainties in yield growth rates associated with different datasets and resolutions were small. The major geographical patterns of yield growth rates derived from the datasets were similar (Fig 3 and Figure F in S1 File). Among the four crops, the recent rate of global mean yield growth was the most rapid for maize and the slowest for wheat (Table 3). These tendencies were common across the datasets and resolutions and are comparable to the reference [5]. However, in the R12 dataset, the yield growth rates of rice (1.5% yr^-1^) were comparable to those of soybean (1.5% yr^-1^), whereas rice yields increased more rapidly (1.7% yr^-1^) than soybean yields (1.5–1.6% yr^-1^) in the I14 dataset regardless of the resolution. These tendencies were different from that given in the reference [5].

**Table 3:** Global average annual yield growth rates.

Crop	% yr^-1^		
	Reference [5]	R12	I14
Maize	1.6 (0.8–2.4)^a^	1.7–1.8	1.8–1.9
Soybean	1.3 (0.3–2.0)	1.5	1.5–1.6
Rice	1.0 (0.5–1.4)	1.5	1.7
Wheat	0.9 (0.1–1.8)	1.3–1.4	1.2–1.4

^a^ 90% confidence limit estimated using the bootstrap method [5].

For both datasets, our estimates of yield growth rate were always more rapid than those of earlier work [5] (Table 3). As a result, anticipated production increases by 2050 estimated using the R12 and I14 datasets were larger than those of the reference [5] but similar to each other (Table 4). We found that the conclusion of earlier work [5] (that recent yield growth rates of the four crops are far lower than the required rates to double their production by 2050 solely from yield growth) is robust against the use of different datasets and resolutions, although uncertainties in absolute terms persistently existed.

**Table 4:** Production increases in 2050 with current yield growth rate.

Crop	% of production in 2008		
	Reference [5]	R12	I14
Maize	67 (34–101)^a^	71–73	76–78
Soybean	55 (13–84)	61–63	64–67
Rice	42 (21–59)	62–63	70–70
Wheat	38 (4–76)	54–57	48–57

^a^ 90% confidence limit estimated using the bootstrap method [5].

### Yield variability changes

The major geographical patterns of yield variability change across the datasets were generally similar (Figure G in S1 File), although different datasets sometimes depicted different yield variability changes. For instance, wheat yield variability in the northeastern part of China increased in the R12 dataset, whereas yield variability in that region decreased in the I14 dataset (the average yield variability over China increased at a rate of 0.003%SD yr^-1^ in the R12 dataset and decreased at a rate of 0.005%SD yr^-1^ in the I14 dataset at 0.083° resolution). Such contrasts across the datasets appeared for maize in the northwestern part of Mexico, soybean in the southeastern part of the US and rice in the northeastern part of China (Figure H in S1 File).

**Fig 4:**
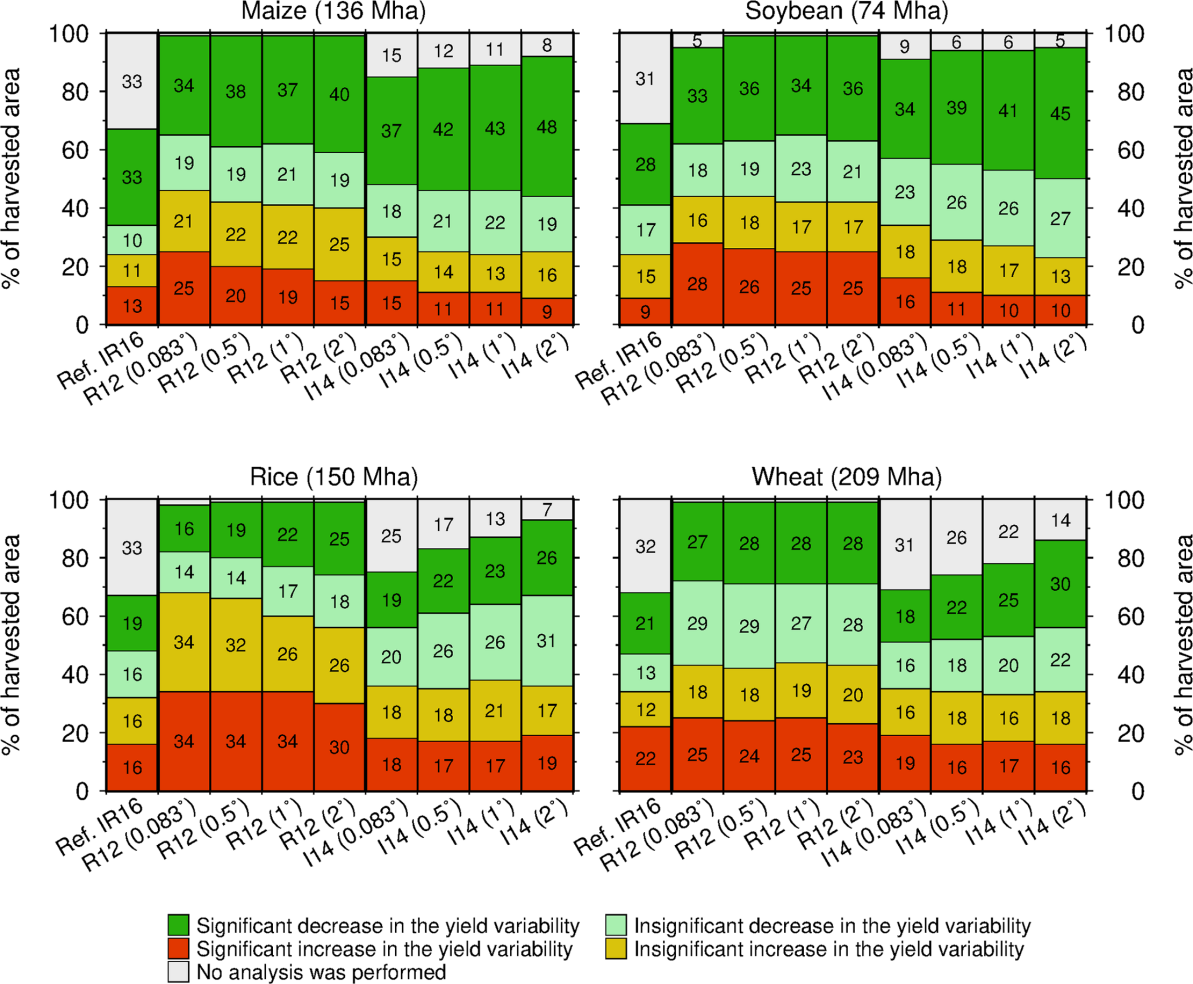
Global harvested area shares by category of yield variability change in 1981–2008 for four crops calculated using the two datasets at four resolutions. The data presented by Iizumi and Ramankutty [6] for 1981–2010 are shown as a reference (IR16). The yield variability changes were divided into the four categories and color coded. The gray shaded area shows that a crop of interest was harvested but yield data are lacking. Area shares less than 2% are not shown.

Different resolutions could lead to contrasting conclusions on yield variability change. The comparison between the 0.083°- and 2°-resolution I14 datasets constituted an extreme case. In the fine-resolution I14 dataset, the areas with contrasting changes in wheat yield variability were comparable: wheat yield variability significantly increased and decreased across 19% and 18% of the harvested area, respectively (Fig 4). In contrast, the coarse-resolution I14 dataset showed that wheat yield variability significantly decreased in many more areas (30%) than where it increased (16%). Similarly, in the 0.083°-resolution R12 dataset, the areas with a significant decrease and increase in the rice yield variability comprised 16% and 34% of the total area, respectively; this led to the interpretation that rice yield variability increased in many more areas that where it decreased. However, in the 2°-resolution R12 dataset, the corresponding values were 25% and 30%, leading to the interpretation that the areas with contrasting changes are comparable. A similar case was found for the maize yield variability change between the 0.083°- and 2°-resolution R12 datasets. As summarized in Table 5, we found that the conclusion of earlier work [6] (that while a decrease in yield variability is the main trend worldwide across crops, yields in some regions of the world have become more unstable) was robust for maize and soybean. For rice and wheat, however, the conclusion on yield variability changes is sensitive to the choice of dataset and resolution.

**Table 5:** Areas with significant yield variability changes.

Crop	% of harvested area in 2000					
	Reference [6]		R12		I14	
	IV^a^	DV^b^	IV	DV	IV	DV
Maize	13	33	15–25	34–40	9–15	37–48
Soybean	9	28	25–28	33–36	10–16	34–45
Rice	16	19	30–34	16–25	17–19	19–26
Wheat	22	21	23–25	27–28	16–19	18–30

^a^ Yield variability significantly increased.

^b^ Yield variability significantly decreased.

## Discussion

**Fig 5:**
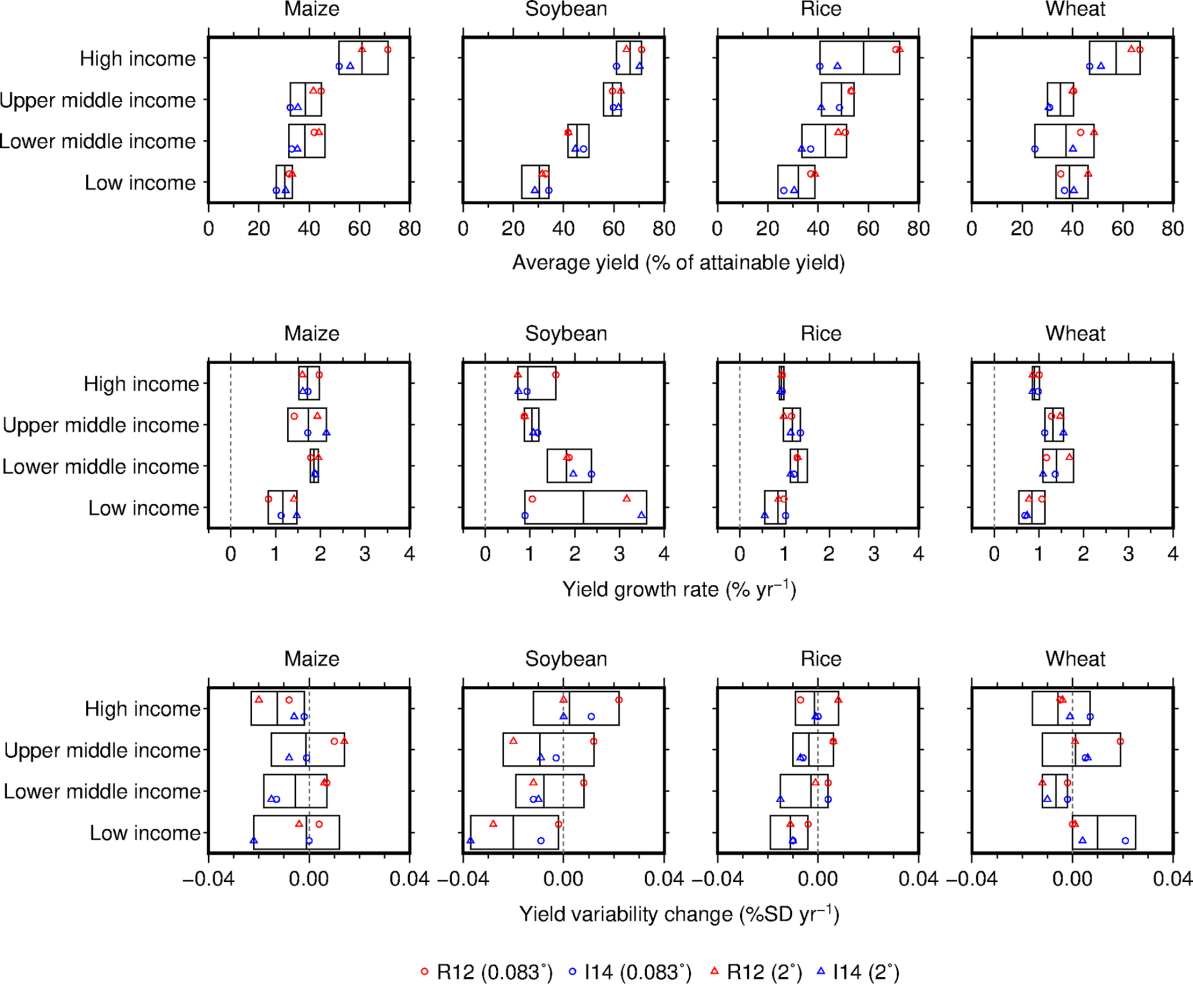
Average yields, growth rates and variability changes of the four crops by income level and their uncertainty associated with different datasets and resolutions. The income level categories in 2016 are based on those of the World Bank [35]. A box indicates the minimum-maximum range. The vertical solid line in the box indicates the average. Data at the finest (0.083°) and coarsest (2°) resolutions are presented to give a sense of which dataset or resolution is a main source of uncertainty.

The correspondence between the datasets at finer resolutions is the highest for yield growth rates, followed by yield gaps and distantly followed by yield variability changes. The close match in yield growth rates at grid-cell levels is not surprising. In the I14 dataset, the main source of information on yield trend is derived from FAO data, which is generally calculated from the subnational data on which the R12 dataset is based. Satellite data used in the development of the I14 dataset contributes to spatial variation in yields within a country more than adjusting yield trends at grid-cell levels. The same reason explains why the datasets match well in terms of yield trend patterns. With high agreement, our results corroborate the reported yield stagnation in some developed countries: wheat in France, Germany and United Kingdom and maize in Italy [15–17]. It is robust that average yield growth in high income countries where current yields are close to the attainable yields is slower than in lower income countries for most cases with exception of maize (Fig 5). It also suggests that yields in low income countries could potentially increase more rapidly at rates observed in upper- and lower-middle income countries.

The differences in estimated grid-cell yield gaps between the datasets are large (Figure C in S1 File). These lead to differences at the country scale, as shown earlier (e.g., rice in Japan). This is likely due to two reasons. One is that the I14 dataset has higher mean values (Figure D in S1 File). The other is an effective difference in the nature of the yield gap calculated based on each dataset. Yields in the R12 dataset are based on average yields of census units, thus the empirical analog method of Mueller et al. [3] approximates the best yield observed in a census unit. The I14 dataset has yields that reflect the grid-cell-scale variability. Thus, application of the method of Mueller et al. [3] to the I14 dataset leads to attainable yields that are an approximation of the best yield observed in a grid cell. Because yields at finer scales can be higher than area-averaged yields [18, 19], it is not surprising that the yield gaps based on the I14 dataset are larger than those based on the R12 dataset. Since the I14 dataset could capture yields at finer scales, it leads to attainable yields closer to an agronomically attainable potential yield in contrast to calculations based on subnational statistics. Which approach is more useful depends on the application–while agronomic potential yield is a useful concept, the factors that lead to high-yielding points at grid scale can not necessarily be translated to a larger scale [20].

The correspondence in estimated grid-cell yield variability changes between the datasets is lower than that in yield growth rates and yield gaps. If we compared the grid-cell yield variability changes of soybean and rice between the 0.083°-resolution datasets, it is found that grid-cell yield variability changes in the I14 dataset varies, whereas those in the R12 dataset are identical (Figure I in S1 File). The yield variability changes across grid cells within a political unit are identical in the R12 dataset, but not in the I14 dataset because yields in the I14 dataset reflect grid-cell-scale variability. Although there is room for further study to better attribute the poor correspondence between the datasets at 0.083° resolution, the spatial aggregation ameliorates this type of error by smoothing the finer-scale spatial variations in a variable of interest and leads to better correspondence between the datasets. Importantly, the benefit of spatial aggregation is consistently found not only for yield variability changes but also yield gaps and yield growth rates. Although the certainty is higher at a country level than the grid-cell level, no clear relationship between yield variability change and income level is found for maize and wheat, although yield variability of soybean and rice more rapidly decreased in lower income countries than in higher income countries (Fig 5).

Our intercomparison highlights the relative advantages and limitations of the individual datasets, which have implications for improving the datasets and ultimately reducing the uncertainties. As already noted, the limited spatial coverage is a limitation of the I14 dataset. This is sourced from crop calendar data used in the algorithm to develop the I14 dataset [9] (i.e., Sacks et al. [21]), which covers only 76–92% of the global harvested area. Although the Monthly Irrigated and Rainfed Cropland Area around the year 2000 data [22] is another major crop calendar data, no separation of growing seasons of a crop (e.g., winter and spring wheat) is available. This precludes its use in the current algorithm. Another limitation of the I14 dataset is the use of time-constant information on harvested area [13]. Sizable differences in regional average yields are observed between use of dynamic and time-constant harvested area [23]. Satellite remote sensing is useful to derive historical map of cropland [24], but still difficult to differentiate areas where a specific crop is grown. The use of time-constant production shares by season in the 1990s [25] is also a limitation of the current algorithm. There are well-documented rationales showing that some regions of the world have experienced drastic change in production shares across seasons (e.g., maize in Brazil [26]).

In contrast, the R12 dataset relies on national and subnational census data, which are a reliable source of information, although there are both quality- and quantity-related problems for some regions, particularly Africa [24]. The R12 dataset covers a longer period than the I14 dataset, but no separation of cropping seasons is available in the R12 dataset.

Our intercomparison reveals that the I14 dataset is capable in capturing spatial variations in yields across grid cells located within a census unit. This leads to an idea that grid-cell yield estimates would become more accurate if subnational yield statistics are used in the algorithm instead of FAO national data. A new hybrid dataset that could be developed by applying the algorithm of Iizumi et al. [9] to the R12 dataset can also incorporate historical change in harvested area into account. Wheat production is commercialized in many regions of the world and there are few subsistence growers compared to other crops with some exceptions in Africa, leading to differences in yield at a finer scale. To address such issues, information on yields in different cropping systems, for instance, irrigated or rainfed and high or low inputs [14], and farm field size [27] might be useful.

There is room for further study. Neither climate-yield relationships nor their historical changes are compared in this study, although earlier studies [6, 28–32] present such assessments using a single dataset. An intercomparison in this regard is worthwhile as a future effort. Different spatial aggregation methods are available to upscale yield data [33, 34] but are not considered here.

## Conclusions

This study presents, for the first time, the uncertainties in estimates of yield gaps, trend patterns, growth rates and variability changes at the global scale associated with different yield datasets and resolutions. The uncertainties vary by variable and crop. The satellite-based I14 dataset indicates larger yield gaps and hence larger potentials for yield improvement than the census-based R12 dataset. Differences in resolution contribute relatively little to the uncertainties in the yield gaps. In contrast, yield variability changes in rice and wheat are sensitive to the choice of dataset and resolution. Yield trend patterns and growth rates are robust across the different datasets and resolutions. Although such uncertainties persist, our analysis indicates that yield gaps in lower income countries are larger than in higher income countries, yield growth rates in middle income countries are higher than in higher and lower income countries, and yield variability changes seem to be more dependent on crop or country than on income level.

## Supporting information

S1 File Supporting information text, table, and figures. (DOCX)
