# Supplementary material for: Uncertainties of potentials and recent changes in global yields of major crops resulting from census- and satellite-based yield datasets at multiple resolutions
Source: PLoS One. 2018 Sep 20;13(9):e0203809. doi: 10.1371/journal.pone.0203809 (PMC6147479; doi:10.1371/journal.pone.0203809)
Supplement: S1 File — (DOCX) [file pone.0203809.s001.docx]

**Supporting information**

**Text A. Updates to the I14 dataset**

The initial version of the I14 dataset covering the period 1982–2006 at 1.125° resolution was developed by Iizumi et al. [9] and updated for the analysis of yield variability change [6] to cover the period 1981–2011 at the same resolution. As done in the previous study [6], we generated grid-cell yield estimates at 0.083° resolution for the present study by applying the algorithm described by Iizumi et al. [9], which combines FAO national yield statistics, satellite-derived crop-specific net primary production (NPP), crop-specific harvested area and crop calendar in 2000 and crop-specific production shares in the 1990s achieved using different cropping seasons.

The updates made by Iizumi and Ramankutty [6] and in this study are summarized in Table A in S1 File. Most importantly, in the updated version of the I14 dataset, grid-cell yields at 0.083° resolution were estimated using the satellite-derived crop-specific NPP data at the same grid resolution, whereas the initial I14 dataset is based on the NPP data at 1.125° resolution (Table A in S1 File). The NPP data in 1981–2011 used in the updated version were calculated based on leaf area index (LAI) and the fraction of photosynthetically active radiation (FPAR) data obtained from the 3^rd^ generation Global Inventory Modeling and Mapping Studies (GIMMS3g) products [37], which were derived by applying a neural network algorithm to the GIMMS3g normalized differential vegetation index (NDVI) data. In contrast, the initial version uses the NPP data in 1982–2006 estimated by applying a relatively simple algorithm described by Los et al. [38] to the NDVI data derived from the NOAA (US National Oceanic and Atmospheric Administration)/AVHRR (Advanced Very High Resolution Radiometer) [39, 40].

Furthermore, handling errors of FAO national yield statistics found in the initial version were adjusted (Table A in S1 File). In the initial version, the grid-cell yield estimates over some countries, such as Democratic Republic of Congo, are missing as a result of handling errors of the FAO data due to the separate and independent of countries, but those errors were fixed in the updated version. Other data, including reanalysis global solar radiation [41], harvested area map in 2000 [13], global crop calendar in 2000 [21] and production shares by cropping season in the 1990s [25], were commonly used between the initial and updated versions.

The generated 0.083°-version I14 dataset was aggregated to derive the coarser-resolution datasets (0.5°, 1° and 2°). The harvested area map in 2000 [13] was used as the weights in the aggregation. Note that the 1.125°-resolution I14 dataset used by Iizumi and Ramanutty [6] was derived from the 0.083°-resolution dataset in the same manner. An important difference between the 1.125°-resolution I14 dataset used in Iizumi and Ramanutty [6] and the 0.083°-, 0.5°-, 1°- and 2°-resolution I14 datasets used in this study is that the 1.125°-resolution dataset is harmonized so that average grid-cell yields in 1997-2003 are close to those of the M3-Crops data [13], while the 0.083°-, 0.5°-, 1°- and 2°-resolution datasets are not.

**References**

1. FAO. FAOSTAT. 2017. Available from: http://www.fao.org/faostat/en/#home.
2. Zhu Z, Bi J, Pan Y, Ganguly S, Anav A, Xu L, et al. Global data sets of vegetation leaf area index (LAI)3g and fraction of photosynthetically active radiation (FPAR)3g derived from global inventory modeling and mapping studies (GIMMS) normalized difference vegetation index (NDVI3g) for the period 1981 to 2011. Rem Sens*.* 2013; 5: 927-948, doi:10.3390/rs5020927.
3. Los SO, Pollack NH, Parris MT, Collatz GJ, Tucker CJ, Sellers PJ, et al. A global 9-yr biophysical land surface dataset from NOAA AVHRR data. J Hydrometeorol. 2000; 1: 183-199, https://doi.org/10.1175/1525-7541(2000)001<0183:AGYBLS>2.0.CO;2.
4. Pinzon J, Brown ME, Tucker CJ. Satellite time series correction of orbital drift artifacts using empirical mode decomposition. In: Huang NE, Shen SSP, editors. Hilbert-Huang Transform: Introduction and Applications, World Scientific, Toh Tuck Link; 2005. pp. 167–186.
5. Tucker CJ, Pinzon JE, Brown ME, Slayback DA, Pak EW, Mahoney R, et al. An extended AVHRR 8-km NDVI data set compatible with MODIS and SPOT Vegetation NDVI data. Int J Rem Sens. 2005; 26: 4485-4498, doi:10.1080/01431160500168686.
6. Onogi K, Tsutsui J, Koide H, Sakamto M, Kobayashi S, Hatsushika H, et al. The JRA-25 reanalysis. Journal of the Meteorological Society of Japan*.* 2007; 85: 369-432, doi:10.2151/jmsj.85.369.

**Table A. A summary of updates made to the I14 dataset.**

|  | Initial version | Updated version |
| --- | --- | --- |
| *General characteristics* | | |
| Reference | Iizumi et al. [9] | Iizumi and Ramankutty [6] and this study |
| Period | 1982–2006 | 1981–2011. Data in 1981 and 2011 are available only for limited locations because of the incomplete cropping season. |
| Resolution | 1.125° (Gaussian grid coordinate system) | 0.083°, 0.5°, 1° and 2° (regular grid coordinate system) for this study. Same as the initial version of Iizumi and Ramankutty [6]. |
| Crops | Maize (major/secondary), soybean, rice (major/secondary), wheat (winter/spring) | Same as the initial version. |
| *Inputs to generate grid-cell yield estimates* | | |
| Crop statistics | National yield statistics [36] | Same as the initial version, but errors in the initial version were fixed (e.g., Democratic Republic of the Congo). |
| Satellite products | 2^nd^ generation GIMMS 0.073° bi-monthly NDVI data [39, 40]. The NDVI data were aggregated to 1.125° using harvested area maps and then used to estimate LAI and FPAR at 1.125° resolution. LAI and FPAR were used to derive crop-specific NPP. | 3^rd^ generation GIMMS 0.083° bi-monthly LAI and FPAR data [37]. Crop-specific NPP at 0.083° resolution was estimated from LAI and FPAR. |
| Global radiation | Onogi et al. [41] | Same as the initial version. |
| Harvested area | Monfreda et al. [13] | Same as the initial version. |
| Crop calendar | Sacks et al. [21] | Same as the initial version. |
| Production share by season | USDA [25] | Same as the initial version. |


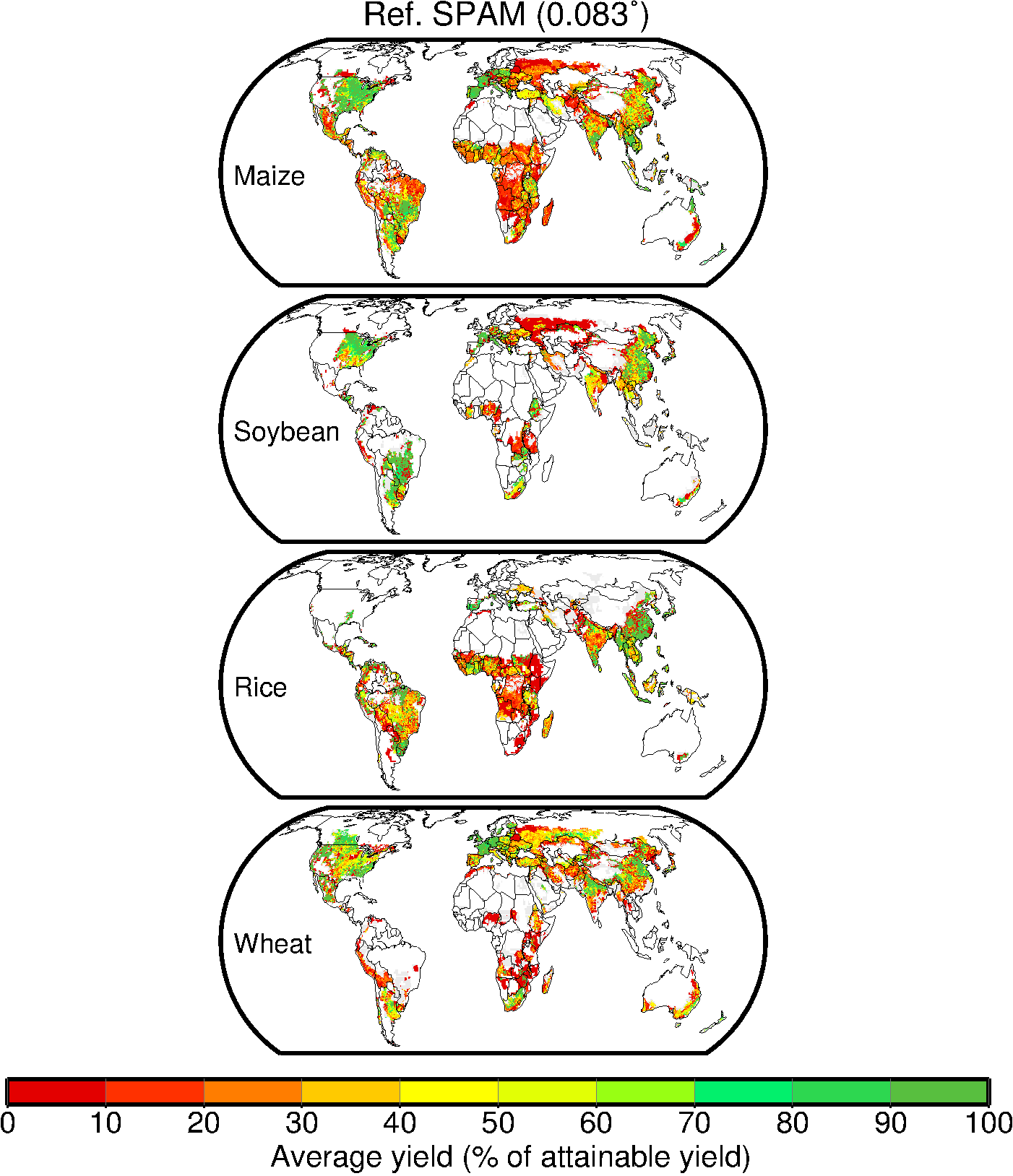


**Figure A. Average yields of the four crops circa the year 2000 (indicated as a percentage of the attainable yield) calculated using the 0.083°-resolution SPAM data.**


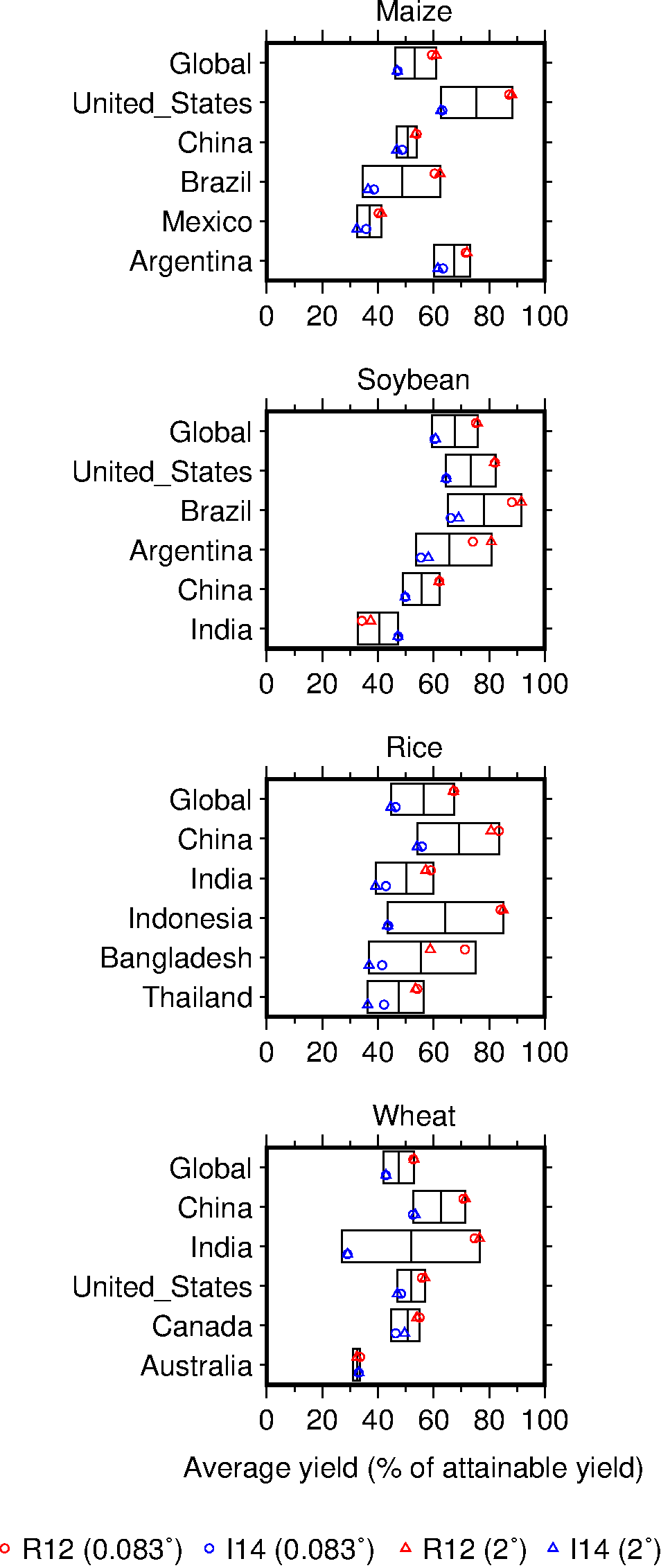


**Figure B. Average yields of the four crops for the globe and for major producers and their uncertainty associated with different datasets and resolutions.** The box indicates the minimum-maximum range consisting of the two datasets and four resolutions. A vertical line in a box indicates the average. Data at the finest and coarsest resolutions are presented to give a sense which dataset or resolution is a main source of uncertainty.


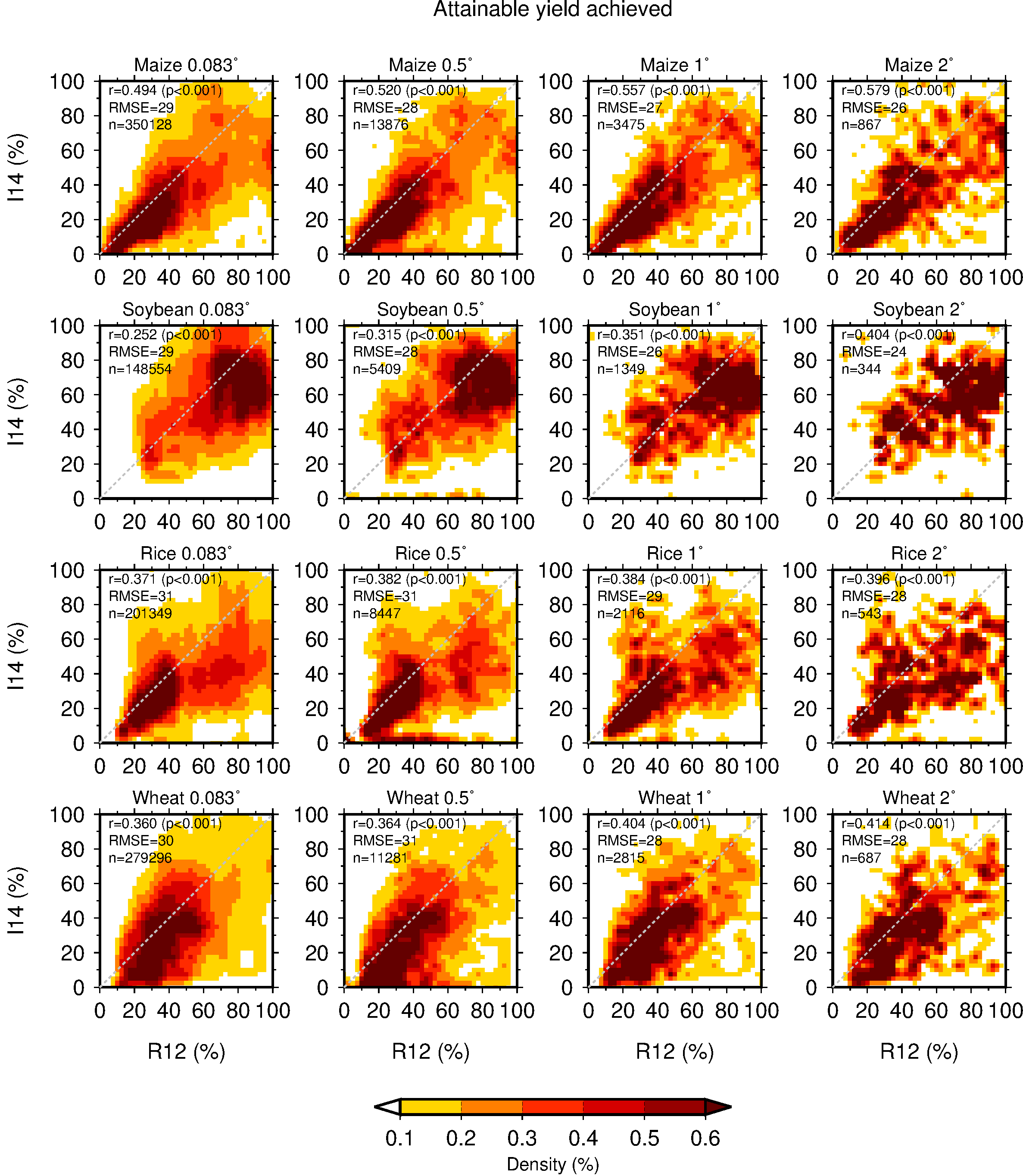


**Figure C. The correspondence in grid-cell average yields for four crops (indicated as a percentage of the attainable yield) calculated using different datasets and resolutions.** The colored shaded area shows the smoothed density of the grid-cell data. The correlation coefficient (r), p-value (p), root-mean-squared error (RMSE) and sample size (n) are presented.


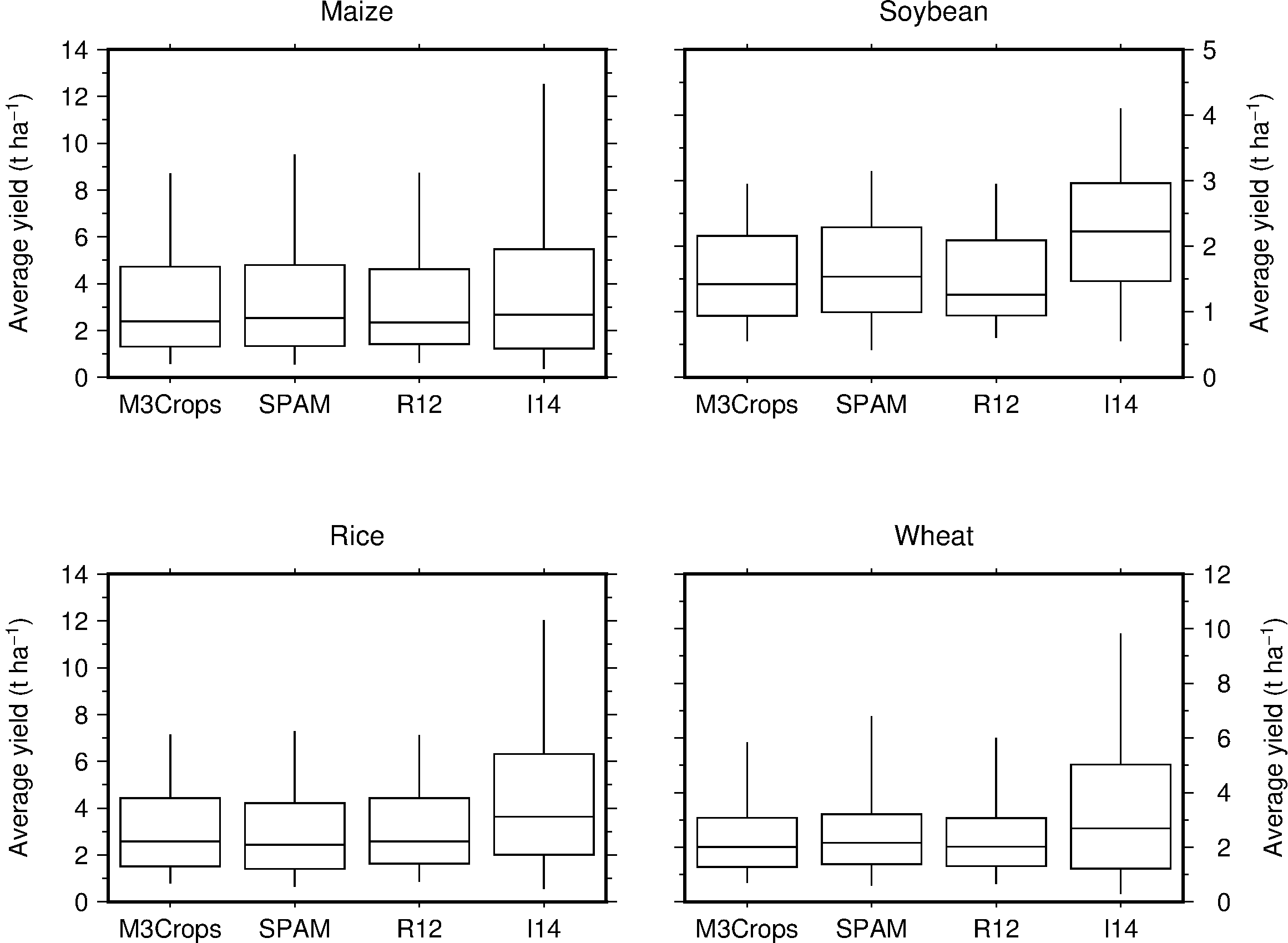


**Figure D. Box plots of grid-cell average yields circa the year 2000 provided using the 0.083°-resolution R12 and I14 datasets as well as M3-Crops and SPAM data.** The horizontal line in a box indicates the median. The lower and upper hinges of a box indicate the 25th and 50th percentiles, respectively. The vertical bar indicates the 90% interval.


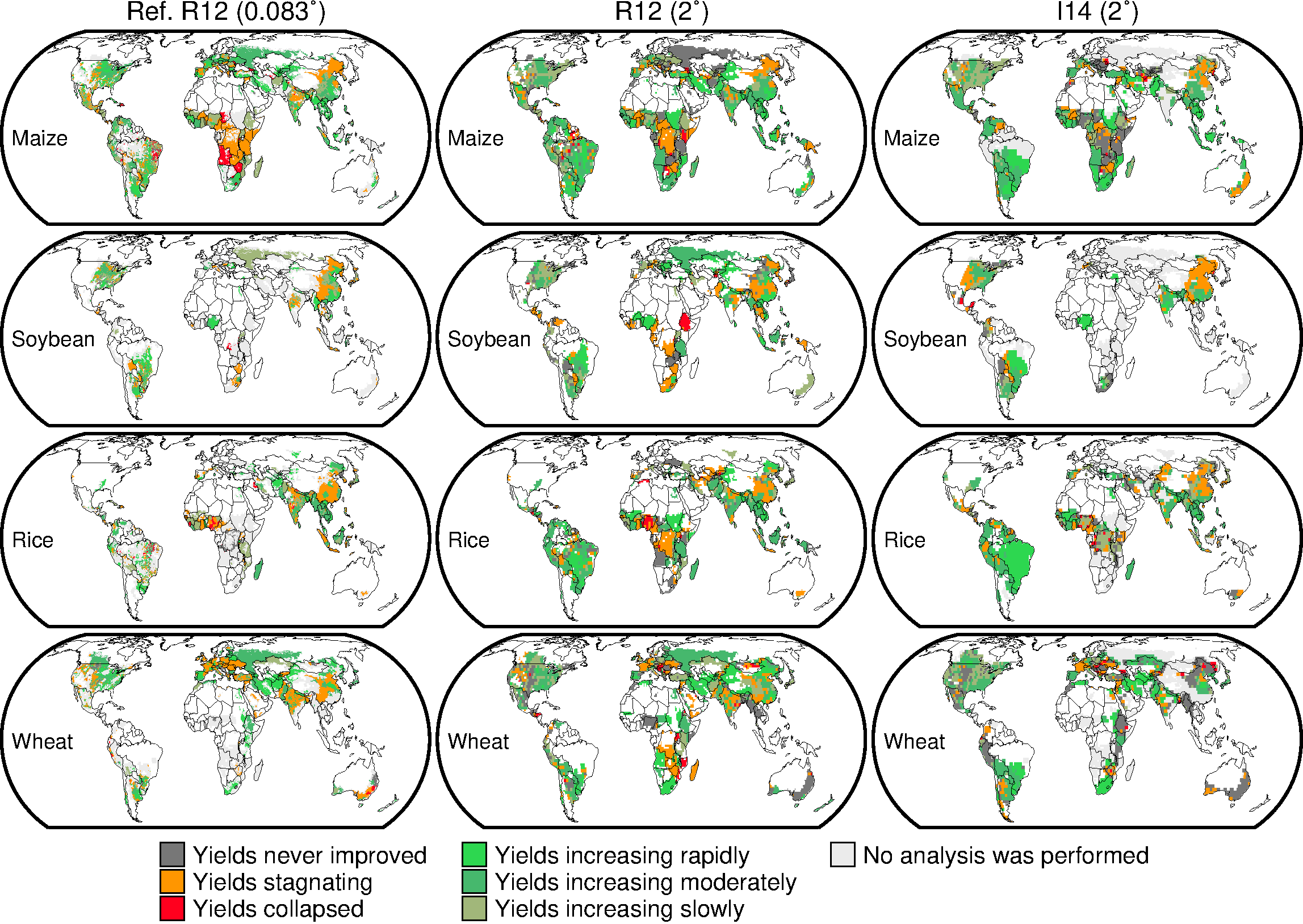


**Figure E. Yield trend patterns for the four crops in 1981–2008 calculated using the two different yield datasets at 2° resolution.** Those presented by Ray et al. [4] are for the period 1961–2008 and are used as a reference (Ref. R12). The data were divided into the six yield trend patterns and color coded. The gray shaded area indicates that a crop of interest was harvested but yield data are lacking.


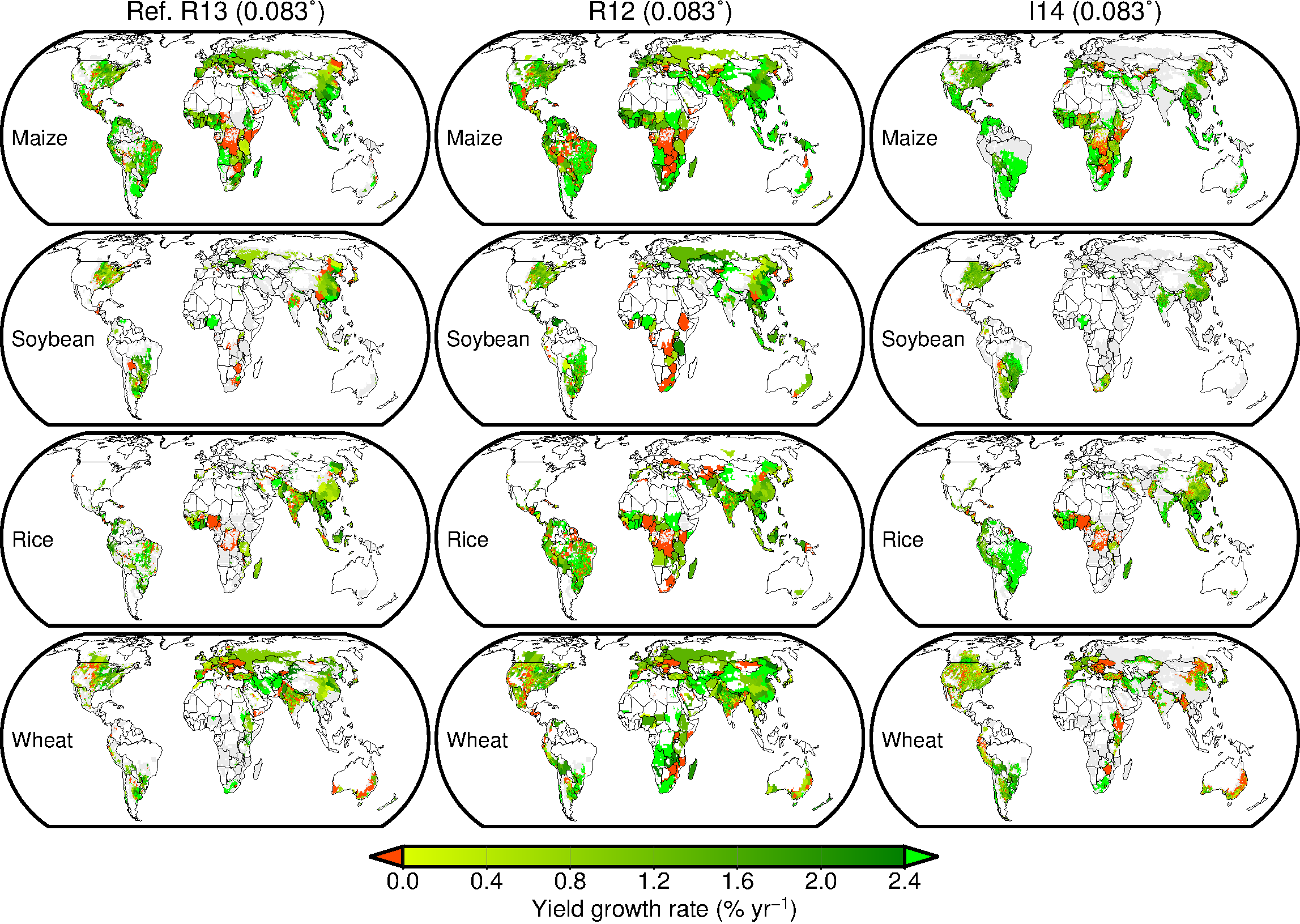


**Figure F. Recent average annual rates of yield growth for the four crops in 1989–2008 computed using different datasets at 0.083° resolution.** Those presented by Ray et al. [5] are shown as a reference (Ref. R13). The red shaded area indicates the areas where yields are declining, whereas the fluorescent green shaded area shows the area where yield growth rates are sufficient to double production by 2050 if sustained. The gray shaded area indicates that a crop of interest was harvested but yield data are lacking.


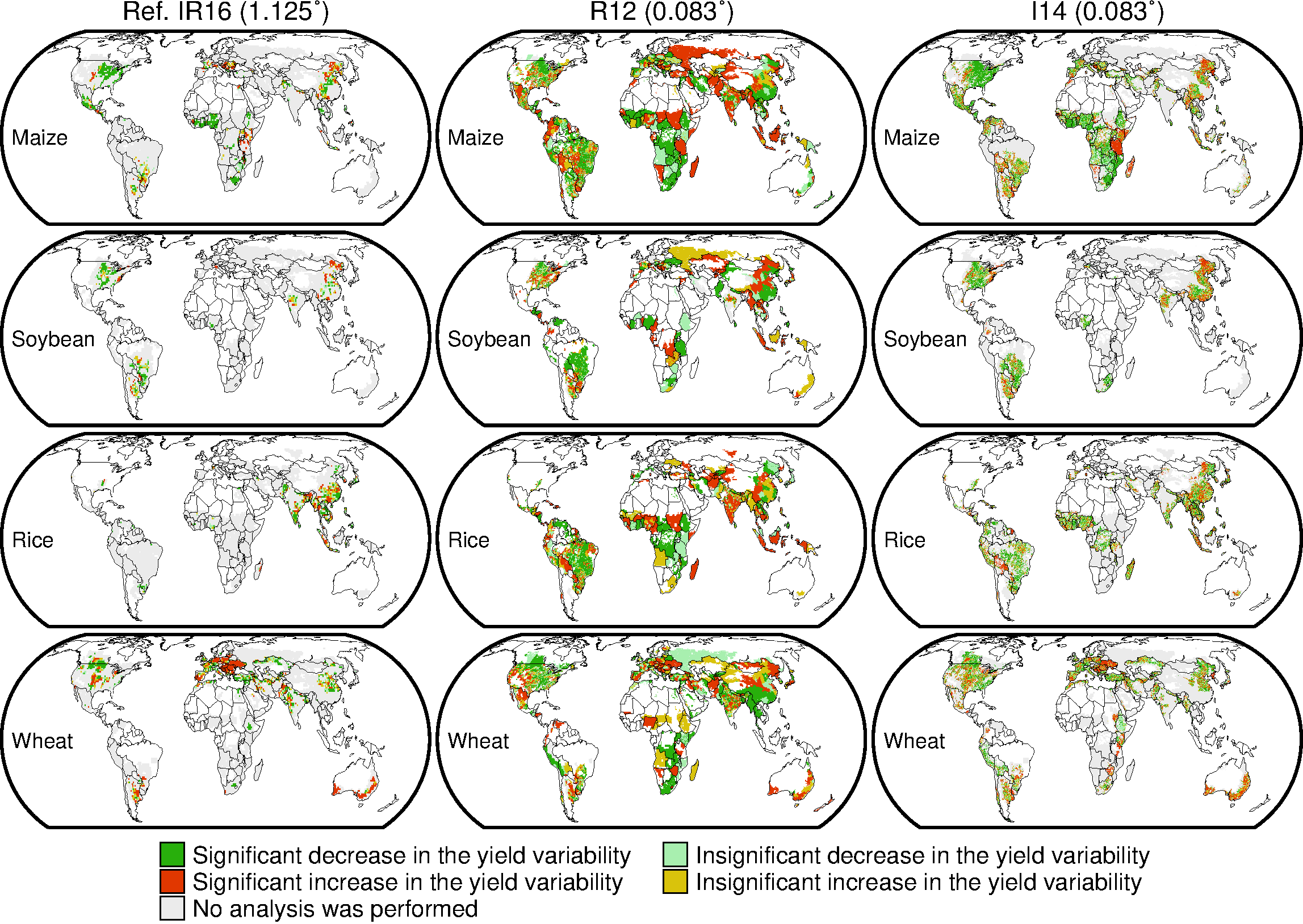


**Figure G.** **Yield variability changes for the four crops in 1981–2008 calculated using different datasets at 0.083° resolution.** Those for the period 1981–2010 presented by Iizumi and Ramankutty [6] are used as a reference (Ref. IR16). The data were divided into the four categories of yield variability change and color coded. The gray shaded area indicates that a crop of interest was harvested but yield data are lacking.


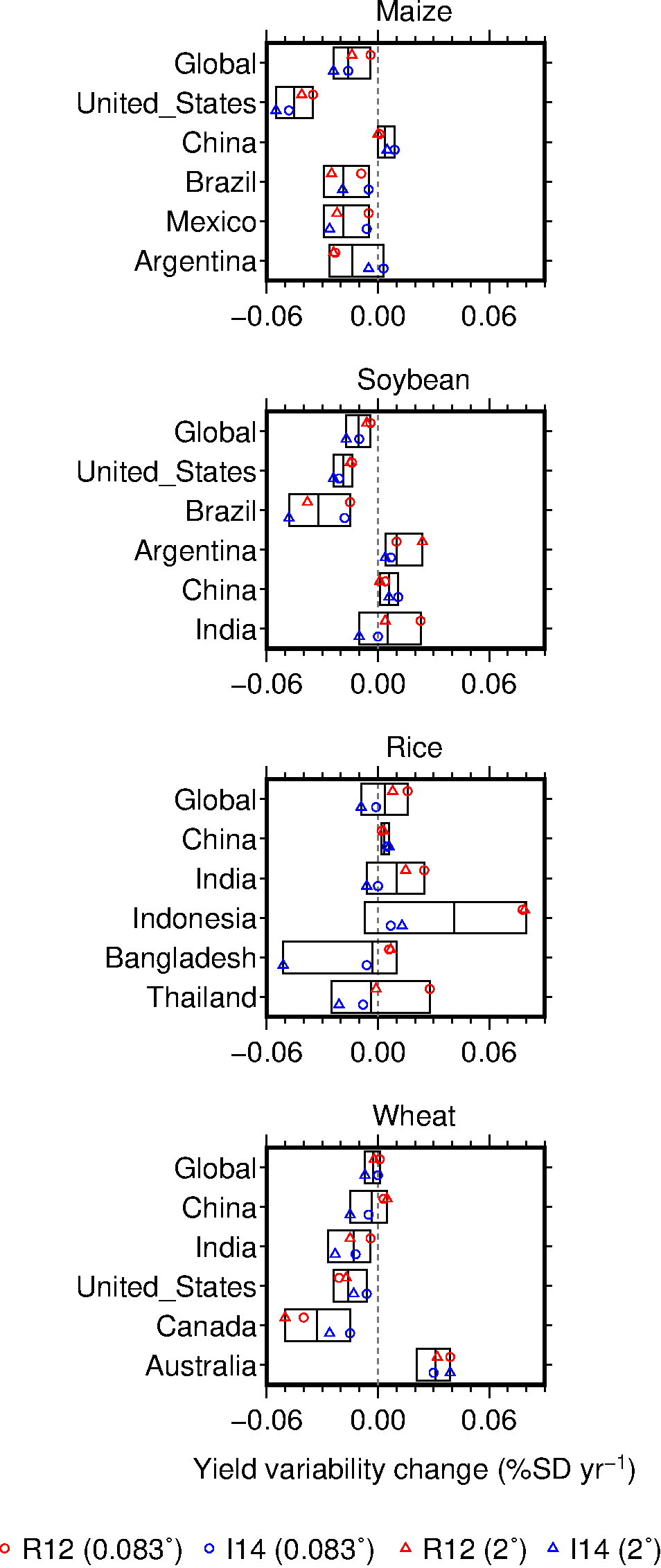


**Figure H.** **Yield variability changes of the four crops in 1981–2008 for the globe and for major producers and their uncertainty associated with different datasets and resolutions.** The box indicates the minimum-maximum range consisting of the two datasets and four resolutions. The vertical line in a box indicates the average. The data at the finest and coarsest resolutions are presented to give a sense of which dataset or resolution is the main source of uncertainty.


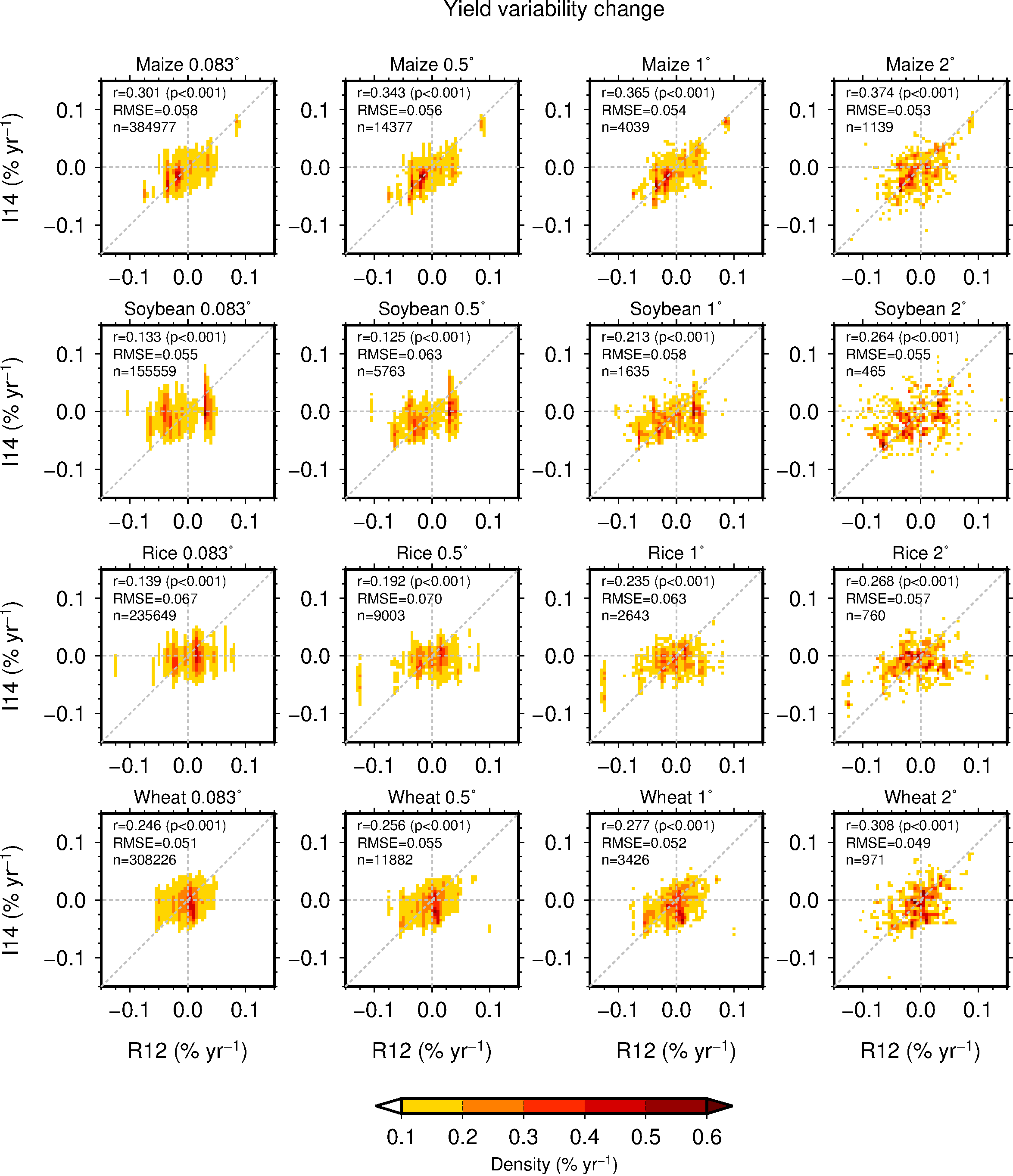


**Figure I.** **The correspondence in grid-cell yield variability changes for the four crops in 1981–2008 calculated using different datasets and resolutions.** The colored shaded area shows the smoothed density of the grid-cell data. The correlation coefficient (r), p-value (p), root-mean-squared error (RMSE) and sample size (n) are presented.
